# Etymologia: Syncytium

**DOI:** 10.3201/eid1904.ET1904

**Published:** 2013-04

**Keywords:** etymologia, syncytium, protoplasm, respiratory illness, respiratory syncytial virus

## Syncytium [sin-sish′e-əm]

From the Greek *syn* (together) and *kytos* (receptacle, vessel), a multinucleate mass of protoplasm produced by the merging of cells. Respiratory syncytial virus was discovered in 1956 by Morris et al., who isolated it from a group of chimpanzees with respiratory symptoms. Morris originally called the new agent “chimpanzee coryza agent,” although when Chanock et al. confirmed that the agent caused respiratory illness in humans, it was renamed because “the striking characteristic of these viruses is the production of syncytial areas in tissue culture.”
